# Development and validation of a risk score to predict adverse birth outcomes using maternal characteristics in northwest Ethiopia: a retrospective follow-up study

**DOI:** 10.3389/fgwh.2024.1458457

**Published:** 2024-12-18

**Authors:** Rahel Mulatie Anteneh, Getayeneh Antehunegn Tesema, Ayenew Molla Lakew, Sefineh Fenta Feleke

**Affiliations:** ^1^ Department of Public Health, College of Health Sciences, Debre Tabor University, Debre Tabor, Ethiopia; ^2^ Department of Epidemiology and Biostatics, Institute of Public Health, College of Medicine and Health Sciences, University of Gondar, Gondar, Ethiopia; ^3^ Department of Public Health, College of Health Sciences, Woldia University, Woldia, Ethiopia

**Keywords:** adverse birth outcome, model, risk score, pregnant women, Ethiopia

## Abstract

**Background:**

Adverse birth outcomes are unfavorable outcomes of pregnancy that are particularly common in low- and middle-income countries. At least one ultrasound is recommended to predict adverse birth outcomes in early pregnancy. However, in low-income countries, imaging equipment and trained manpower are scarce. According to our search of the literature, there is no validated risk prediction model for predicting adverse birth outcomes in Ethiopia. Hence, we developed and validated a model and risk score to predict adverse birth outcomes using maternal characteristics during pregnancy for use in resource-limited settings.

**Methods:**

A retrospective follow-up study was conducted from 1 January 2016 to 31 May 2021, and a total of 910 pregnant women were included in this study. Participants were selected using a simple random sampling technique. Stepwise, backward multivariable analysis was conducted. The model's accuracy was assessed using density plots, discrimination, and calibration. The developed model was assessed for internal validity using bootstrapping techniques and evaluated for clinical utility using decision curve analysis across various threshold probabilities.

**Results:**

Premature rupture of Membrane, number of fetuses, residence, pregnancy-induced hypertension, antepartum hemorrhage, hemoglobin level, and labor onset remained in the final multivariable prediction model. The area under the curve of the model was 0.77 (95% confidence interval: 0.73–0.812). The developed risk prediction model had a good performance and was well-calibrated and valid. The decision curve analysis indicated the model provides a higher net benefit across the ranges of threshold probabilities.

**Conclusion:**

In general, this study showed the possibility of predicting adverse birth outcomes using maternal characteristics during pregnancy. The risk prediction model using a simplified risk score helps identify high-risk pregnant women for specific interventions. A feasible score would reduce neonatal morbidity and mortality and improve maternal and child health in low-resource settings.

## Background

Pregnancy outcomes can be positive or negative (adverse) (1). Adverse birth outcomes (ABO) are undesirable outcomes of pregnancy, which include low birth weight (LBW), birth asphyxia, small for gestational age, congenital anomalies, sepsis, intrauterine growth restriction (IUGR), preterm birth, and admission to the neonatal intensive care unit (2–4). Stillbirth, LBW, and preterm birth are the most common adverse pregnancy outcomes in low- and middle-income countries (LMICs), including Ethiopia (5–7).

Adverse birth outcomes are indicators of maternal health during pregnancy and at birth (8). Maternal health and nutritional status before and during pregnancy determine pregnancy outcomes (9). Globally, an estimated 210 million women become pregnant each year, and over 75 million pregnancies end in stillbirth or preterm birth (10). The literature shows that congenital anomalies, LBW, preterm birth, and maternal complications are responsible for 50.9% of infant deaths (11).

The chances of a newborn surviving, growing, maintaining long-term health, and developing psychosocially can all be predicted based on birth outcomes (12). The majority of the survivors experience frequent infections, sleep apnea, persistent lung diseases, anemia, jaundice, and respiratory distress (13). Adverse birth outcomes are also associated with increased fetal and neonatal morbidity and mortality, chronic disease in life, and long-term physical and psychological problems (14), and are strong biological predictors of developmental outcomes (15, 16). They also impose significant social and economic consequences on the family (17).

Adverse birth outcomes are significant public health problems and a leading cause of under-5 morbidity and mortality worldwide. The problem is highly prevalent in developing countries, including Ethiopia. Across the globe, an estimated 15 and over 20 million newborns are born preterm or with low birth weight, respectively (15, 16, 18). More than one million of these babies died shortly after birth, and countless others suffered from lifelong physical, neurological, psychological, and educational disabilities (15).

The most frequently reported causal factors linked to adverse birth outcomes include medical conditions and nutritional status of the mother or fetus, genetic influences, environmental exposure; infertility treatments, behavioral, socioeconomic and socio-demographic factors, and past and recent obstetric conditions (15, 19–22). Underlying maternal medical conditions and poor nutrition during pregnancy are responsible for 1.4 million stillbirths and 98% of perinatal mortalities in low- and middle-income countries (10, 23, 24). These are the most preventable and treatable contributors to adverse birth outcomes (5).

Government policies in recent years have emphasized maternal and child health by increasing input into the health system (25). Following the failure to achieve the Millennium Development Goal (reduction of under-5 mortality by two-thirds), a sustainable development goal (SDG) was established. The SDG targets the “end of preventable deaths of newborns and children under five, with all countries aiming to reduce neonatal mortality at least as low as 12 per 1,000 live births and under-five mortality as low as 25 per 1,000 live births” (26). According to the Ethiopia Demographic Health Survey (EDHS) report, neonatal mortality in Ethiopia showed a slight decline (from 39% to 33%) between 2005 and 2019 (27).

Various studies have been conducted on the prevalence of adverse birth outcomes and their contributing factors. However, according to our review of the literature, there are scarce studies on the forecasting of unfavorable Ethiopian birth outcomes. Numerous researchers have also recommended further research to identify novel ways to manage third-trimester pregnant women in low-resource settings who are more likely to have an unfavorable pregnancy outcome (28).

Risk prediction models for adverse birth outcomes have been developed in high-resource settings. In a study conducted to develop a risk score model for predicting the risk of adverse birth outcomes, the discriminative power of the area under the receiver operating characteristics curve (AUROC) was 0.79 and the cutoff point was 20%, with a sensitivity of 29%, specificity of 82%, and positive and negative predictive values of 22% and 85%, respectively (29). A Ugandan study found that it was impossible to predict adverse perinatal outcomes, and the model performance AUROC was 0.66 (30). However, in low-resource contexts, such as Ethiopia, the predictors they utilized, such as laboratory markers, are not typically carried out. Therefore, it is crucial to create a risk prediction model for adverse birth outcomes in the context of our nation, Ethiopia. As a result, our goal was to develop and validate a model for predicting the likelihood of unfavorable delivery outcomes in pregnant women who attend antenatal care (ANC) visits at the Comprehensive and Specialized Hospital of the University of Gondar (UOGCSH). Therefore, researching the risk of adverse birth outcomes in pregnant women plays a crucial role in diagnosis, prediction, and the creation and implementation of effective interventions among high-risk populations. To ensure positive outcomes, it is beneficial for healthcare professionals to plan, launch, and intervene with healthcare services early rather than late during an emergency. It aids in the planning and delivery of quality healthcare services by policymakers. In addition, by recognizing high-risk mothers and offering them care, it is possible to lower the prevalence of poor newborn outcomes, preventable neonatal illness, and mortality at the local, regional, national, and worldwide levels.

## Methods

### Study aim, design, and setting

The aim of the research was to develop and validate a model for predicting the likelihood of unfavorable delivery outcomes in pregnant women who attend ANC visits at the UOGCSH. A retrospective follow-up study design was applied. The hospital is one of the largest tertiary-level teaching and referral hospitals in Ethiopia and is found in Gondar Town. Gondar is located 737 km away from Addis Ababa, the capital city of Ethiopia. The study was conducted from 1 January 2016 to 21 May 2021. The hospital serves as a referral center for 7 million people in the administrative zone and the residents in the surrounding areas. Gynecology and Obstetrics is one of the major departments in the School of Medicine in the hospital. Antenatal care and delivery services are among the services provided in the hospital at no cost. During the study period, 15,680 women received prenatal care at UOGCSH. There were 9,040 pregnant women who received antenatal care and gave birth there in total. The hospital has 20 senior gynecologists, 58 residents, and 178 midwives.

### Study variables and data collection procedure

Adverse birth outcome (Yes or No) was the dependent variable for this study. The independent variables were socio-demographic characteristics (maternal age, marital status, and place of residence), antenatal characteristics [time of initiating ANC visit, hemoglobin (HGB) level during pregnancy, pregnancy status, amniotic fluid index, number of ANC visits, antepartum hemorrhage (APH), premature rupture of membranes (PROM), pregnancy-induced hypertension (PIH), gestational diabetes mellitus (GDM), number of the fetuses, presentation, and lie of the fetus], medical conditions [urinary tract infection (UTI), human immune virus (HIV), chronic hypertension, and other comorbidities], and labor and delivery characteristics (duration of labor, onset of labor, and mode of delivery).

After receiving ethical approval from the University of Gondar's Institutional Review Board (IRB) and consent from the UOGCSH's medical director and midwifery department head, the data were accessed from the records. We could not obtain consent from study subjects because it was secondary data. From 20 March to 21 May 2021, eight BSc midwives collected data by evaluating charts from individual records using a structured data extraction checklist and were supervised by two recruited MSc midwives and a primary investigator.

### Operational definition

**ABO:** Women who had at least one of the following birth outcomes: stillbirth, low birth weight, preterm birth, and congenital anomaly (31).
**Preterm birth:** Defined as the birth of a neonate with a gestational age above 28 weeks but below 37 weeks (20).
**Congenital anomaly:** Refers to when a newborn is recorded as having any body parts with congenital defects (32).
**Stillbirth**: A baby born with no signs of breathing after weeks gestation (31)
**Low birth weight:** Defined as a birth weight less than 2,500 g (33).

### Data processing and analysis

The collected data were cleaned and cross-checked for completeness and consistency. The data were entered into Epi-Data version 4.6 statistical software and exported to STATA version 14 and R version 4.05 for analysis. Since missing data occurred at random, multiple imputations were used to fill up the gaps. Hemoglobin level, family planning, marital status, the start of labor, amniotic fluid index, and pregnancy status were among the predictors that were imputed, with 34 (3.7%), 20 (2.2%), 13 (1.4%), 13 (1.4%), 87 (9.6%), and 16 (1.8%) missing values respectively. The variance inflation factor was used to check for multicollinearity among the independent predictors.

To identify the predictors of adverse birth outcomes, a binary logistic regression model was used. The multivariable binary logistic regression analysis took into account variables from the bivariate binary logistic regression analysis with *p*-values <0.25. Using the log-likelihood ratio test >0.15, a stepwise backward multivariable analysis was performed starting with the full model to obtain the final reduced model. Given that the prognostic model was similar to a full multivariable model for the outcome variable, a *p*-value cutoff value of 0.15 was created to prevent the rejection of potentially significant variables in the absence of confounding adjustment.

### Risk prediction model development and validation for ABO

The developed risk prediction model was used to predict the risk of adverse birth outcomes. The occurrence relationship was established based on the identified predictor variables:

Probabilityofadversebirthoutcome=f(predictorvariables)

Riskpredictionwithscore=β1residence+β2APH+β3PIH+β4PROM+β5HGB+β6N_Fetus+β7onsetlabor

The determinants were PIH, APH, PROM, number of fetuses, hemoglobin level, the onset of labor, and residence, and the domain was pregnant women.

The significant factors from the final reduced model were used to create a simple risk score that served as the foundation for the risk prediction model. The coefficients were divided by the lowest coefficient and rounded to the nearest integer for the purpose of creating easily usable adverse birth outcome risk prediction scores from the findings of the multivariable binary logistic regression

Finally, total risk scores were determined by adding up the scores of each variable effect for individuals (29, 34). The Youden index was used to estimate the cutoff point for the predicted probability of an adverse birth outcome and risk stratification based on the developed clinical prediction model (35). Sensitivity analysis was conducted on the different cutoff points to classify low-risk and high-risk pregnant women. Sensitivity, specificity, negative predictive value, and positive predictive value were calculated for the clinical performance evaluation of the model (36).

The model performance was evaluated by its discrimination and calibration ability. Calibration characterized model performance in terms of the agreement between predicted and observed risk for adverse birth outcomes using the “givitiR” package in “R” software. The calibration ability of the model was statistically evaluated, if it was significant it had good predictive ability. This was depicted graphically with a calibration plot with a 45° inclination at the intercept of 0 and slope 1 [ratio of observed and expected equal to one (O/E = 1)] as the best possible calibration (29).

Discrimination (ability to discriminate adverse outcomes from no adverse outcomes) was evaluated based on AUROC with a 95% confidence interval (CI). A ROC curve is used to assess the performance of the categorical classifiers using the “ROCit” and “pROC” packages in R software with the plot of sensitivity (true positive rate) vs. 1 − specificity (false positive rate). Bootstrapping with 10,000 samples was resampled with replacements from the dataset with complete predictors to assess the internal validation. A decision curve for our model was included to aid clinical decision-making based on a risk threshold preference (37) for public health impact and clinical utility of the developed model. Finally, the results were presented using statements, tables, and figures and reported according to the transparent reporting of a multivariable prediction model for individual prognosis or diagnosis (TRIPOD) statement (38).

## Results

### Baseline socio-demographic, reproductive history, and medical characteristics of the study participants

A total of 910 pregnant women who had an ANC visit were included in this study, with a response rate of 98.5%. The median age of the mothers was 26 years, with an interquartile range (IQR) of 22–30 years. The majority (746, 81.98%) of the mothers were between the ages of 20 and 34 years. More than three-fourths (809, 88.90%) of the mothers were urban residents, and 868 (95.38%) mothers were also married. Furthermore, 93 (10.22%) participants had medical illnesses. Of these, 59 (63.4%) and 31 (33.3%) mothers had urinary tract infections and were seropositive for HIV, respectively (Tables 1–3).

**Table 1:** Socio-demographic characteristics of the pregnant women who had ANC visits at UOGCSH, Ethiopia, 2016–2020 (*n* = 910).

Variable	Category	Frequency	Percentage
Maternal age	<20	66	7.25
	20–34	746	81.98
	≥35	98	10.77
Residence	Urban	809	88.90
	Rural	101	11.10
Current marital status	Unmarried	42	4.62
	Married	868	95.38

The unmarried marital status includes pregnant women who had not married (had no husband) during their most recent pregnancy and includes single, widowed, and divorced women. Married refers to women who had a husband during their most recent pregnancy.

**Table 2:** Maternal reproductive history and birth characteristics of the pregnant women who had ANC visits at UOGCSH, 2016–2020.

Variable	Category	Frequency	Percentage
Gravidity	Primigravida	351	38.57
	Multigravida	559	61.43
Parity	Zero	399	43.85
	Primiparity	229	25.16
	Multiparity	282	30.99
Past obstetric outcome related to the fetus	Abortion (*n* = 559)	106	18.9
	Stillbirth (*n* = 511)	23	4.5
	Early neonatal death (*n* = 511)	25	4.89
	Low birth weight (*n* = 511)	2	0.4
	Preterm (*n* = 511)	2	0.4
Modern family planning use	No	430	47.25
	Yes	480	52.75
Type of family planning used	Injectable	252	52.5
	Pills	156	32.5
	Implanon	63	13.12
	Others^a^	9	1.87

^a^ Others include family planning such as emergency contraceptives and an intrauterine contraceptive device.

**Table 3:** Medical illness of the pregnant mother who had ANC visits at UOGCSH, 2016–2020.

Variable	Category	Frequency	Percentage
Medical illness	No	817	89.78
	Yes	93	10.22
Type of medical illness	HIV/AIDS	31	33.3
	UTI	59	63.4
	Chronic hypertension	5	5.4
	Others	20	21.5

HIV/AIDS, human immune deficiency virus/acquired immune deficiency syndrome; UTI, urinary tract infection.

Other types of medical illnesses include hepatitis B, cardiac illness, kidney disease (nephrolithiasis), thyrotoxicosis, hypothyroidism, and diabetes mellitus.

### Recent antenatal characteristics, pregnancy complications, and labor and delivery characteristics

In this study, 491 (53.96%) of the mothers initiated ANC visits in the second trimester of their pregnancy. Nearly two-thirds (571, 62.5%) of the mothers had four or more ANC visits for their most recent pregnancy. Furthermore, 65 (7.14%) had low hemoglobin levels and 857 (94.2%) women had pregnancies that were planned and wanted. Moreover, 202 (22.2%) of the mothers had recent pregnancy complications, of which 74 had PIH (36.6%), 67 had PROM (33.2%), and 57 had APH (27.2%). In total, 888 (97.58%) pregnant women had singleton pregnancies and 866 (95.16%) had a normal presentation (vertex). A majority, (816, 89.67%) of the mothers initiated their labor spontaneously and 305 (33%) mothers had prolonged labor (Tables 4,5).

**Table 4:** Antenatal characteristics of the pregnant women in their recent pregnancy who attended the ANC unit at the UOGCSH, 2016–2020.

Variable	Category	Frequency	Percentage
Maternal hemoglobin level (g/dl)	Normal	835	91.76
	Low	75	8.24
Timing of ANC initiation	1st trimester	77	8.46
	2nd trimester	491	53.96
	3rd trimester	342	37.58
Number of ANC visits	1	53	5.82
	2–3	286	31.43
	≥4	571	62.75
Iron folate supplementation	No	89	9.78
	Yes	821	90.22
Time of initiating iron folate supplementation	1st trimester	12	1.46
	2nd trimester	385	46.78
	3rd trimester	426	51.76
Pregnancy status	Planned and wanted	857	94.18
	Unplanned but wanted	30	3.30
	Unplanned and unwanted	23	2.53
Nutritional advices	No	376	41.32
	Yes	534	58.68
Rh factor	Positive	840	92.31
	Negative	70	7.69

ANC, antenatal care; Rh factor, rhesus factor.

**Table 5:** Recent pregnancy complications and labor and delivery characteristics of the pregnant women who had ANC visits at UOGCSH, 2016–2020.

Variable	Category	Frequency	Percentage
Recent pregnancy complication	No	708	77.80
	Yes	202	22.20
Type of recent pregnancy complication	PIH	74	36.6
	APH	57	28.2
	PROM	67	33
	Others	83	41
Number of fetuses	Singleton	888	97.58
	Multiple	22	2.42
Presentation of fetus	Normal	866	95.16
	Malpresentation	44	4.84
Onset of fetus delivery	Induced	94	10.33
	Spontaneous	816	89.67
Mode of delivery	Spontaneous	634	69.7
	Cesarean section	226	24.84
	Instrumental	50	5.49

APH, antepartum hemorrhage; PIH, pregnancy-induced hypertension; PROM, premature rupture of membrane; GDM, gestational diabetic mellitus.

Other recent pregnancy complications include amniotic fluid index (oligohydramnios and polyhydramnios) and chorioamnionitis. Malpresentation presentation of fetus is a presentation other than vertex.

### Incidence of adverse birth outcome

The incidence of adverse birth outcomes was 21.43% (95% CI: 18.79–24.2), of which approximately 14.29% had one adverse birth outcome. The most common adverse birth outcomes were low birth weight (12.42%), preterm delivery (11.87%), stillbirth (5.16%), and congenital anomaly (1.65%). Anencephaly and hydrocephalus are the most common congenital anomalies (Table 6).

**Table 6:** The incidence of adverse birth outcomes among pregnant women who had ANC visits at UOGCSH from 2016 to 2020.

Birth outcome	Category	Number	Percentage
Adverse birth outcome	No	715	78.57
	Yes	195	21.43
Status of newborn	Live birth	863	94.84
	Stillbirth	47	5.16
Birth weight (g)	<2,500	113	12.42
	≥2,500	797	87.58
Gestational age at the time of delivery	Preterm	108	11.87
	Term	802	88.13
Congenital malformation	No	895	98.35
	Yes	15	1.65

Preterm birth is birth before 37 completed weeks; congenital malformation denotes visible congenital defects including anencephaly, encephalocele, hydrocephalus, ventricular defect, cleft pallet, and clubbing of the foot.

### Model development and validation

The demographic, medical, recent antenatal care and related conditions, delivery, and labor characteristics of the mothers were considered for the development of a prediction model for adverse birth outcomes. Underlying medical illness, PROM, number of fetuses, residence, PIH, APH, hemoglobin level, gestational diabetes mellitus, prolonged labor, and presentation had a *p*-value of equal or less than 0.25 in the bivariable binary logistic regression analysis. However, underlying medical illness, prolonged labor, presentation (Table 7), and GDM were excluded from the multivariable binary logistic regression analysis using the likelihood ratio test at a *p*-value >0.15. The goodness of fit test for the fitted model resulted in an insignificant value of 0.999 (Table 8).

**Table 7:** The univariable analysis for adverse birth outcomes among the pregnant women who had ANC visits at UOGCSH, 2016–2020.

Variable	Coefficient with 95% CI	*p*-value
Age		
20–34	0.19 (−0.45 to 0.84)	0.567
>35	0.43 (−0.34 to 1.21)	0.273
Marital status (married)	0.023 (−0.77 to 0.82)	0.954
Residence	1.1 (0.63 to 1.50)	<0.001
Medical illness (yes)	0.63 (0.16 to 1.1)	0.008
Family planning history (no)	0.10 (−0.215 to 0.417)	0.533
Pregnancy status (unplanned)	0.049 (−0.61 to 0.71)	0.883
Time initiating ANC (reference: 1st trimester)		
Second trimester	−0.22 (−0.80, 0.34)	0.437
Third trimester	0.017 (−0.56, 0.60)	0.954
Number of ANC visits (reference: ≥4)		
1 time	0.20 (−0.45 to 0.85)	0.551
2–3 times	0.039 (−0.306 to 0.38)	0.823
Gravidity (multigravida)	0.033 (−0.29 to 0.36)	0.840
GDM (yes)	0.096 (1.20 to 1.39)	0.004
AFI (reference: normal)		
Oligohydramnios	0.087 (−0.74 to 0.56)	0.794
Polyhydramnios	0.45 (−0.91 to 1.81)	0.516
APH (yes)	1.8 (1.29 to 2.405)	<0.001
PIH (yes)	2.0 (1.52 to 2.526)	<0.001
PROM (yes)	1.5 (1.03 to 2.05)	<0.001
Hemoglobin (low)	1.30 (0.820 to 1.79)	<0.001
Number of fetuses (multiple)	2.13 (1.21 to 3.04)	<0.001
Presentation (abnormal)	1.19 (0.57 to 1.80)	<0.001
Labor onset (induced)	1.35 (0.913 to 1.796)	<0.001
Mode of delivery (reference: SVD)		
Instrumental	0.106 (−0.82 to 0.61)	0.770
Cesarean section	0.057 (−0.43 to 0.31)	0.761
Prolonged labor (yes)	0.247 (0.081 to 0.576)	0.040

CI, confidence interval; SVD, spontaneous vaginal delivery; AFI, amniotic fluid index.

**Table 8:** Multivariable binary logistic regression coefficients and risk scores for variables retained in the final reduced model for the prediction of adverse birth outcomes among the pregnant women attending the ANC at UOGCSH, 2016–2020.

Predictor	Adverse birth outcome		Multivariable analysis		Simplified risk score
	Yes	No	*β* (95% CI)	*p*-value	
Residence					
Urban	154	655	0	0.00028	1
Rural	41	60	0.937 (0.42–1.43)		
APH					
No	161	692	0	0.0002719	2
Yes	34	23	1.651 (1.024–2.28)		
PIH					
No	149	687	0	0.00108	2
Yes	46	28	1.761 (1.198–2.33)		
HGB level					
Normal	160	675	0	0.00957	2
Low	35	40	1.372 (0.82–1.92)		
PROM					
No	160	683	0	0.0000148	2
Yes	35	32	1.295 (0.70–1.88)		
N_Fetus					
Singleton	168	710	0	0.00036	3
Multiple	17	5	2.551 (1.5–3.73)		
Onset of labor					
Spontaneous	151	665	0		
Induced	44	50	0.8184 (0.28–1.34)	0.00232	1
Intercept			−2.23876		13 total risk score

APH, antepartum hemorrhage; PIH, pregnancy-induced hypertension; HGB, hemoglobin; PROM, premature rupture of membranes; N_Fetus, number of fetuses during recent pregnancy; CI, confidence interval.

For the simplified risk score we divided the coefficient of the predictors in the final reduced model to the lowest coefficient, which was 0.8184, and rounded to the nearest integer.

### Risk prediction model development

The model was created using the original beta coefficient and a simplified risk score. The calibration and discrimination of the developed model were evaluated. Discrimination (the capacity to distinguish between a bad outcome and no negative consequence) was evaluated using the AUROC with a 95% CI. The final model we examined was more capable of discrimination.

### AUROC and calibration

**Figure 1:**
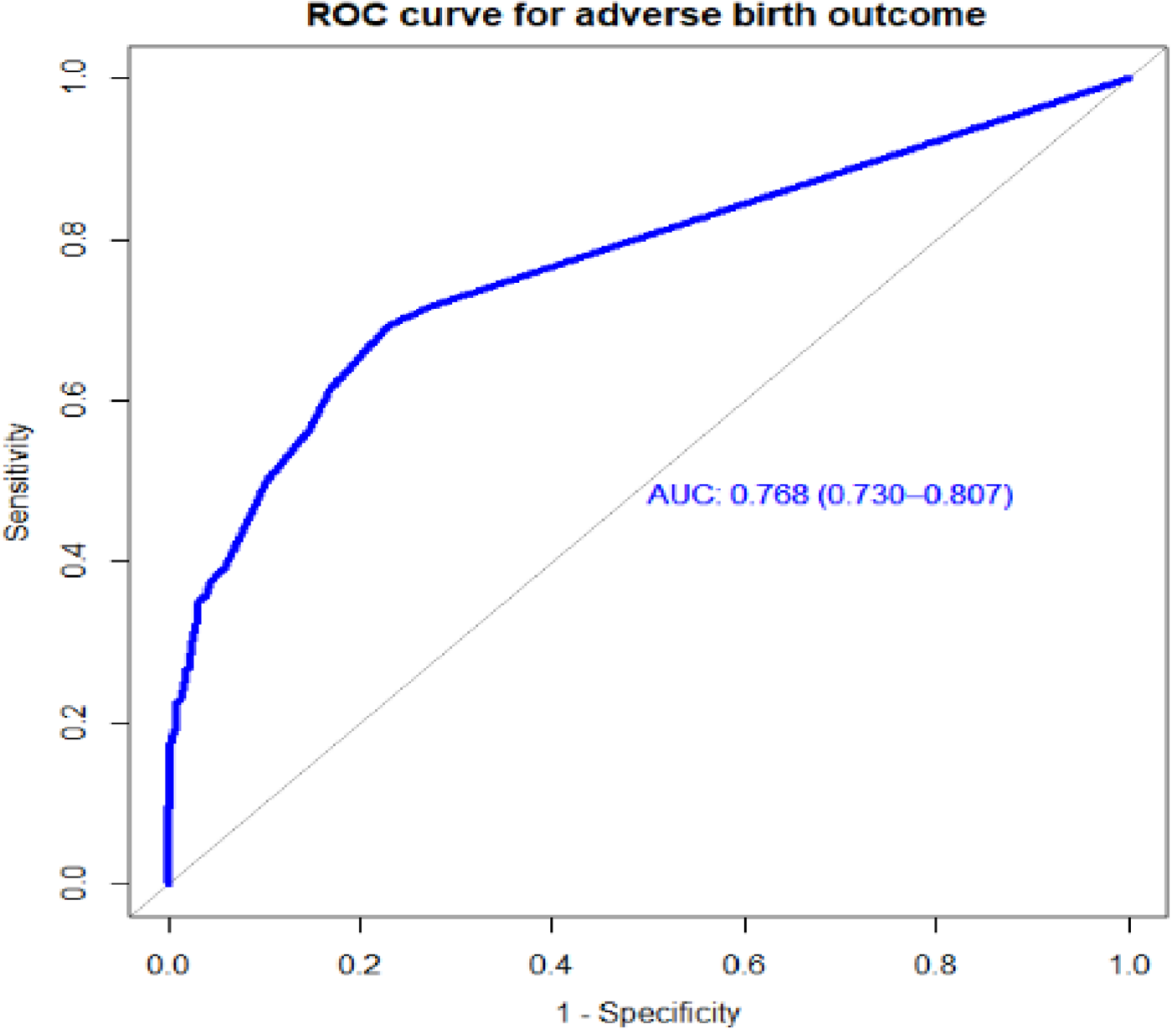
The ROC curve represents the probability of risk for adverse birth outcomes among women who had ANC visits at UOGCSH, 2016–2021.

Individual predictors in the final reduced model performed poorly in identifying pregnant women at risk for adverse birth outcomes, with AUROC values ranging from 0.57 to 0.65. However, when combined, they showed good discrimination. When the APH, PROM, and hemoglobin level AUROC values were combined, the predictive performance was 0.69 (95% CI: 0.64–0.72); when APH, PIH, HGB, and PROM were combined, it was 0.72 (95% CI: 0.67–0.76). Finally, the area under the ROC of the final reduced model using seven predictors was 76.85% (95% CI: 72.7%–80.97%) using the original beta coefficients (Figure 1). The linear predictors using the beta coefficient were calculated as follows:

$$\begin{array}{rl} & \mathrm{Linear}\;\mathrm{predictors}\;\mathrm{estimated}\;\mathrm{risk}\;\mathrm{of}\;\mathrm{ABO}=1/(1+\exp-(-2.24)\\ & \;+0.937\times \mathrm{residence}(\mathrm{rural})+1.65\times \mathrm{APH}(\mathrm{yes})\\ & \;+1.76\times \mathrm{PIH}(\mathrm{yes})+1.37\times \mathrm{HGB}\;(\mathrm{low})+1.29\times \mathrm{PROM}(\mathrm{yes})\\ & \;+2.55\times N\_\mathrm{Fetus}(\mathrm{multiple})+0.818\times \mathrm{labor}\;\mathrm{onset}(\mathrm{induced})) \end{array}$$

**Figure 2:**
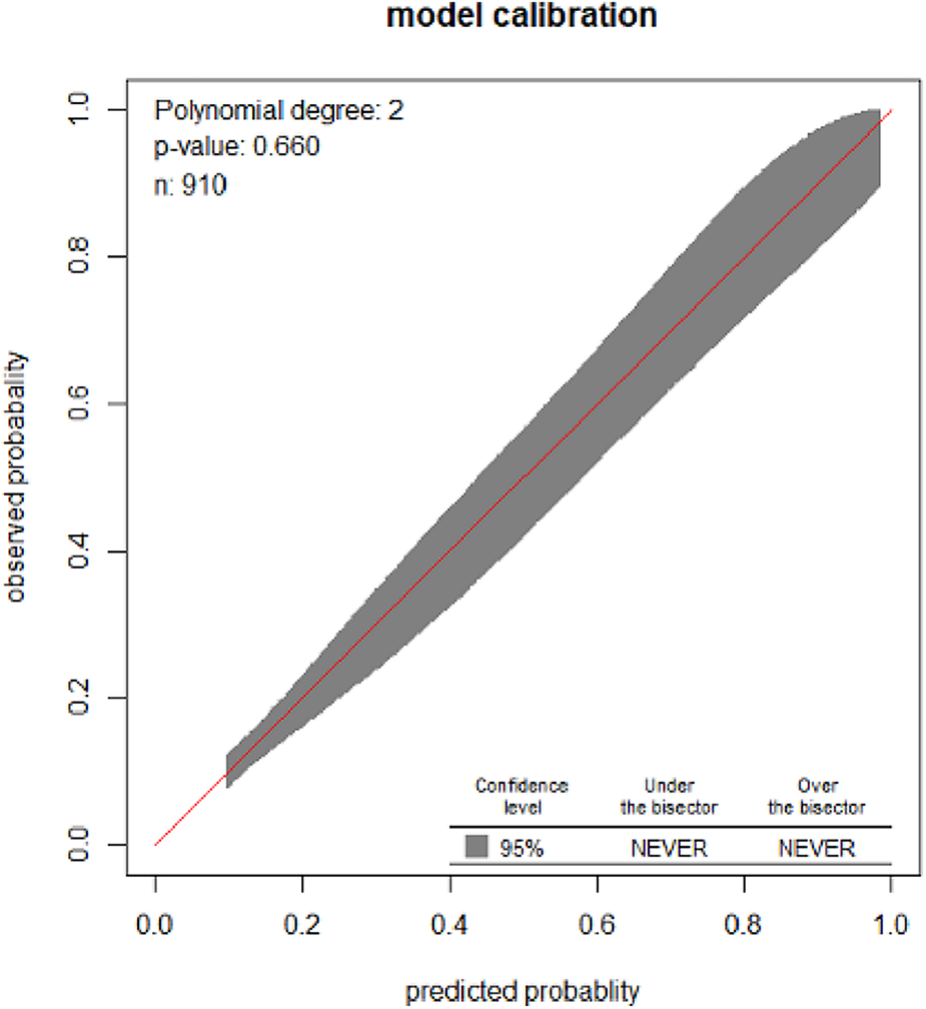
Calibration plot for the developed model based on the original beta coefficients for the risk prediction model for pregnant women who received ANC at UOGCSH, 2026–2021.

The developed model was well-calibrated (*p* = 0.66), which indicates that the model well represented the data (there was agreement between the observed outcomes and the predicted probability) (Figure 2).

### The cutoff point for the probability of ABO

**Figure 3:**
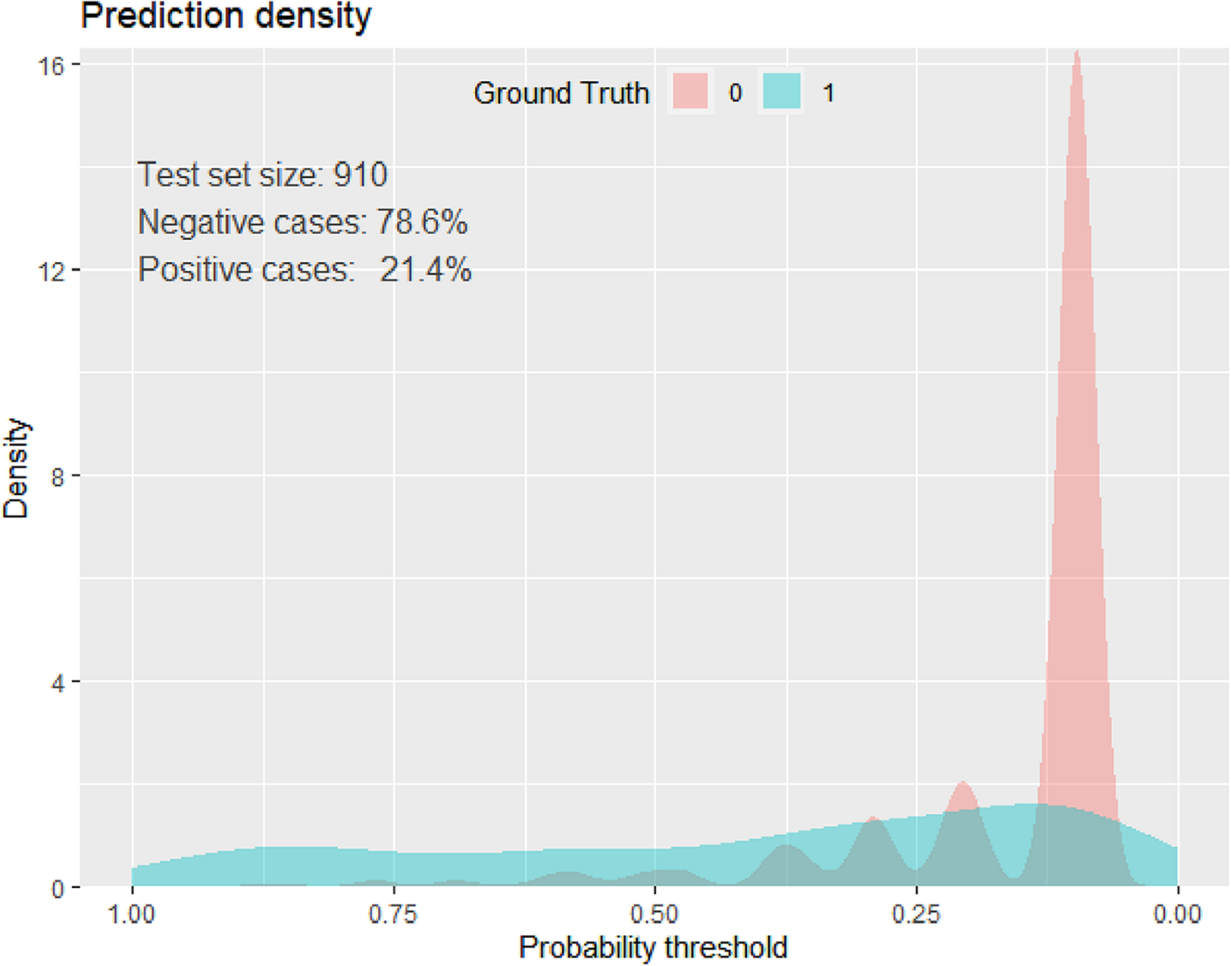
Prediction density plot for developed model using original beta coefficients at UOGCSH, 2016–2020.

The best cutoff point for the predicted probability of an adverse birth outcome using the beta coefficients of the predictor variables was 0.2139 (Table 9), and the specificity, sensitivity, negative predictive, and positive predictive values were, respectively, 83.9%, 67.54%, 90%, and 53%. In addition, the accuracy was 83.3%, and the kappa was 0.4 (Figure 3).

**Table 9:** Performance of the prediction model based on the original beta coefficients at different cutoff points for the pregnant women who received ANC at UOGCSH, 2016–2020.

Cutoff point	Sensitivity	Specificity	PPV	NPV	Accuracy
0.1536	76	74	44	92	74
0.2000	70	80	48	90.8	77
0.2139	67.54	83.9	53	90	80
0.416	50.2	94	70	87	84

### Prediction model development using simplified risk scores

**Figure 4:**
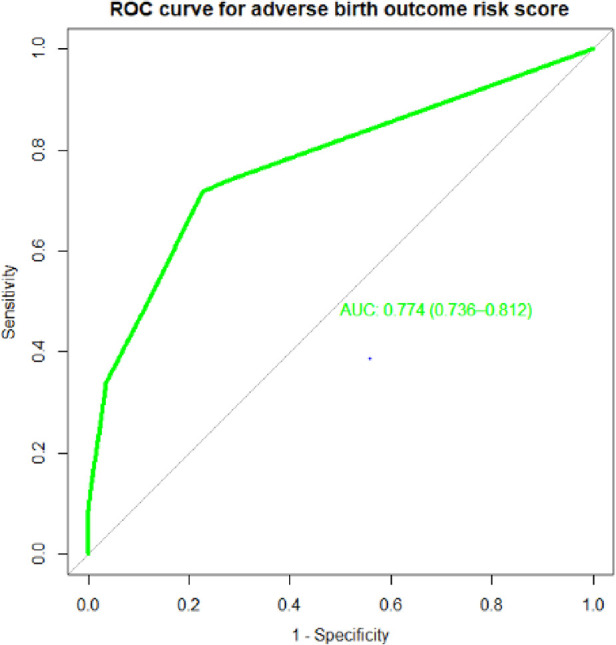
Area under the ROC curve for the prediction model for ABO using simplified risk score.

The final reduced model's significant regression coefficients were all employed to construct a model with a streamlined risk score. The calibration had a *p*-value of 0.782, and the AUROC for the condensed risk score prediction model was 77% (95% CI: 73%–81.2%) (Figure 4). For the development of the prediction model, we selected the simplified risk score over the original beta coefficient since it was simpler, needed little adjustment, and was simple to apply.

$$\begin{array}{rl} & \mathrm{Simplified}\;\mathrm{risk}\;\mathrm{score}\;(\mathrm{ABO})=1\times \mathrm{residence}(\mathrm{rural})+2\times \mathrm{APH}(\mathrm{yes})\\ & \;+2\times \mathrm{PIH}(\mathrm{yes})+2\times \mathrm{HGB}(\mathrm{low})+2\times \mathrm{PROM}(\mathrm{yes})\\ & \;+3\times N\_\mathrm{Fetus}(\mathrm{multiple})+1\times \mathrm{labor}\;\mathrm{onset}(\mathrm{yes}) \end{array}$$

### Model validation

**Figure 5:**
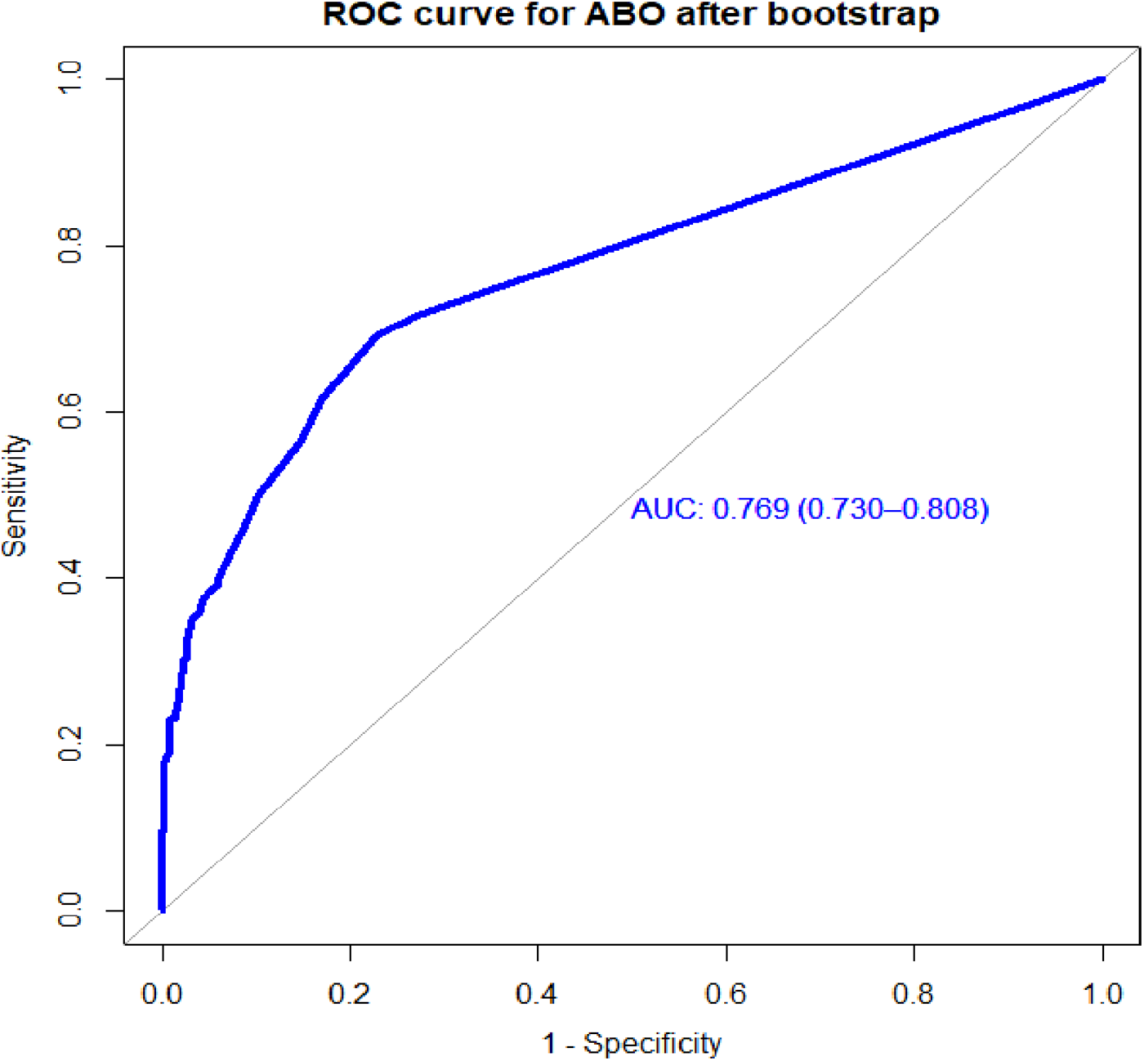
Area under the ROC curve for the prediction model for the bootstrapped sample, UOGCSH, 2016–2021.

The bootstrapping technique was employed to validate the model to check for overfitting and bias. Based on the bootstrap dataset coefficients, the AUROC was evaluated while taking the risk score and bias into account. Actual performance minus expected performance, which was 0.01, was also used to construct the optimism-corrected estimate. The optimistic coefficient (pooled bias) for the model with the coefficient result was 0.009137, and the area under the curve (AUC) was 0.769 (95% CI: 0.7299–0.8076). The model's performance was 0.769 (95% CI: 0.730–0.808), after optimism was corrected for (Figure 5).

### Risk classification for ABO using a simplified risk score

We created a simplified risk score from the model using all the regression coefficients in the reduced model for easy practical utility. The model developed using the risk score outperforms the model developed using the original beta coefficient, with an AUC of 0.77.4 (95% CI: 0.73.6–0.812). The minimum and maximum possible scores were 0 and 13. The proportion of adverse birth outcomes in the group of pregnant women at low-risk (score 2) was 55 (9.04%), there were 74 (35.1%) in the intermediate-risk (scores 2–4) group, and 66 (72.5%) in the high-risk group (score 4) (Table 10).

$$\begin{array}{rl} & Risk\;score=1\times \mathrm{residence}\;(\mathrm{rural})+(2\times \mathrm{APH}\;(\mathrm{yes}))+\\ & \;(2\times \mathrm{PIH}(\mathrm{yes}))+(2\times \mathrm{hemoglobin}(\mathrm{low}))+(2\times \mathrm{PROM}(\mathrm{yes}))+\\ & \;(3\times N\_\mathrm{Fetus}(\mathrm{multiple}))+(1\times \mathrm{labor}\;(\mathrm{induced})) \end{array}$$

**Table 10:** Risk classification for adverse birth outcome based on the simplified risk scores (*n* = 910) among the pregnant women who received ANC at UOGCSH, 2016–2020.

Risk score category	Prediction model based on maternal characteristics	
	Total number of women	Incidence of ABO
Low risk (<2)	608 (66.8%)	55 (9.04%)
Intermediate (2–4)	211 (23.18%)	74 (35.1%)
High risk (≥4)	91 (0.1%)	66 (72.5%)
Total	910 (100%)	195 (21.43%)

Risk score = 1 × residence(rural) + (2 × APH (yes)) + (2 × PIH(yes)) + (2 × hemoglobin(low)) + (2 × PROM(yes)) + (3 × N_Fetus(multiple)) + (1 × labor (induced)).

The optimal cutoff point was suggested by the Youden index, and thus, pregnant women who scored 2 were classified as low-risk, and pregnant women who scored 2 and above were classified as having a higher risk of an adverse birth outcome. When we dichotomized high-risk (2) and low-risk (2) based on the risk scores, 302 (33.2%) pregnant mothers were at high risk and 608 (66.8%) pregnant mothers were at low risk for an adverse birth outcome. The sensitivity, specificity, positive predictive, and negative predictive values of the risk scores at the optimal cutoff value were 71.79%, 77.79%, 46.4%, and 90%, respectively, with a positive likelihood ratio of 3.16 and a negative likelihood ratio of 0.36 (Table 11).

**Table 11:** Performance of the risk score at different cutoff points for adverse birth outcomes among the pregnant women who attended the antenatal care unit at UOCSH from 2016 to 2020.

Cutoff	High risk *n* (%)	Sensitivity	Specificity	PPV	NPV	LP+	LR−
1	338 (37.1)	73.8	72.8	42.6	91.08	2.71	0.359
2	302 (33.18)	71.79	77.34	46.35	90.95	3.16	0.365
3	184 (20.2)	50.25	87.97	53.26	86.6	4.177	0.56
4	91 (10)	33.84	96.5	72.52	84.24	9.666	0.686
5	66 (7.25)	25.12	97.62	74.24	82.70	10.55	0.76

PPV, positive predictive value; NPV, negative predictive value, LR+, positive likelihood ratio; LR−, negative likelihood ratio (60, 61).

### Decision curve analysis

**Figure 6:**
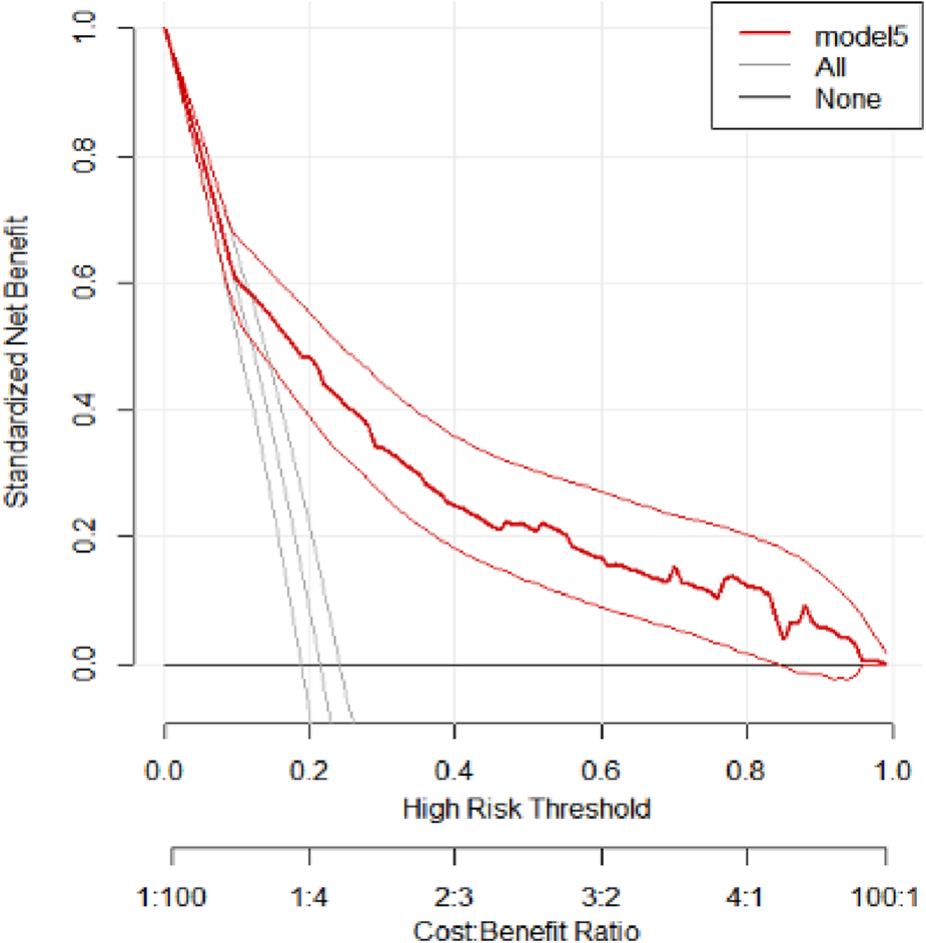
Decision curve analysis of the developed model. The net benefit vs. threshold probability between the model and two extreme scenarios at UOGCSH, 2016–2021, is plotted.

The AUC, sensitivity, specificity, and accuracy were used to assess the model's performance, but decision curve analysis (DCA) was used to assess the model's clinical and public health utility. Using a probability threshold of greater than 0.15, the developed model (Model 5) demonstrates the highest net benefit compared to two alternative strategies: referring all individuals regardless of risk (represented by the horizontal black line) and referring none (illustrated by the vertical line close to the red line). The model has a similar net benefit to those referring to all patients regardless of their risk across thresholds ranging from 0 to 0.15 (Figure 6).

## Discussion

The incidence of adverse birth outcomes was 21.43% (95% CI: 18.79%–24.2%). The most common adverse birth outcomes were low birth weight (12.42%), preterm birth (11.87%), stillbirth (5.16%), and congenital anomaly (1.65%). The incidence of adverse birth outcomes in this study was high even when we excluded the referred pregnant women from the study population (39). However, the overall and individual outcome incidences in Ethiopia are consistent with findings from other systematic reviews and meta-analyses: 26.88% (32) and 23% (15) in Gondar. The finding in this study was lower than in studies conducted in North Wollo, with 31.8% (32), and Gondar, with 23% (15). The finding in this study was also lower than studies conducted in North Wollo, with 31.8% (40), Dessie, with 32.5% (31), and Brazil, with 41.8% (41). However, it was higher than findings from Hawassa, with 18.5% (42), and Kembata Tembaro, with 13.5% (43). This disparity might be due to differences in the study period and design, place of delivery, quality of maternal service utilization, and population included in the study (43).

The combined maternal characteristics used to predict the risk of an adverse birth outcome were residence, antepartum hemorrhage, pregnancy-induced hypertension, premature rupture of membranes, hemoglobin level during pregnancy, multiple gestations, and the onset of labor.

In this study, pregnancy-induced hypertension was a significant predictor for adverse birth outcomes; women who had pregnancy-induced hypertension were at increased risk for an adverse birth outcome. The finding was supported by studies conducted in Tigray, Dessie, China, Iran, and Zimbabwe (2, 31, 44–46). Pregnancy-induced hypertension puts women at a higher risk for organ failure and cardiovascular disorders, both of which affect maternal and fetal health outcomes (47).

Premature rupture of membranes also had a significant association with adverse birth outcomes. Women who had premature rupture of membranes are more likely to have an adverse birth outcome than women who do not. The finding is supported by studies conducted in Kembata in the southern part of Ethiopia, and Debre Tabor (43, 48). This could be due to amniotic fluid, which is necessary for fetal movement and lung expansion and may influence birth outcomes (49).

Low hemoglobin (anemia) during a recent pregnancy was also linked to a poor birth outcome. Similarly, studies conducted in Gondar, Hosanna, and Tigray (10, 15, 50) reported that anemia was significantly associated with adverse birth outcomes. Similarly, this finding is also consistent with findings from Ghana and Pakistan (6, 51). This might be due to the poor nutritional status of the mother and not having sufficient meals during pregnancy, which might cause micronutrient deficiency during pregnancy that has been shown to have serious implications for fetal outcomes. As a result, mothers with anemia or low hemoglobin were more likely to have an adverse birth outcome.

Multiple gestation was a significant predictor for adverse birth outcomes. The finding was consistent with studies conducted in Brazil (29, 52, 53), and it also had a predictive effect when combined with other predictors according to a study by Medicaid in the United States of America (29). Having multiple gestation causes pregnancy complications that cause unfavorable maternal and fetal outcomes. This implies that all requirements (such as nutrition) during a multiple gestation pregnancy are greater than during a singleton pregnancy.

An antepartum hemorrhage in a recent pregnancy was a significant predictor of having an adverse birth outcome. This finding was consistent with previous research, which found that having an antepartum hemorrhage was associated with a higher risk of adverse birth outcomes than not having an antepartum hemorrhage (40, 54, 55).

Induced labor was associated with a poor birth outcome. Pregnant women who were induced were more likely to have an adverse pregnancy outcome than women who went into labor naturally. The finding was consistent with a study from Australia that showed that the induction of labor was associated with an adverse pregnancy outcome (56, 57).

The WHO recommends at least one ultrasound for every pregnant woman before 24 weeks of gestation to estimate fetal outcomes (58, 59). This retrospective follow-up study was conducted to develop a prediction model for adverse birth outcomes for pregnant women attending an antenatal care visit at UOGCSH using maternal characteristics. Even though a risk prediction model cannot replace ultrasound for assessing adverse birth outcomes, we can apply it in clinical settings that do not have access to ultrasound or other invasive procedures or investigations.

Individual predictors with discriminating ability in pregnant women for risk of an adverse birth outcome performed poorly, but their combined effect performed well. The final model had good discriminative power, with an AUROC of 0.768 based on the coefficients, and when the internal validity bias was corrected for the performance was 0.769. When based on the risk score, the performance of the predictive model was 0.774. When using the simplified risk score, its ability to discriminate between pregnant women at higher and lower risk for adverse birth outcomes was 77%.

We prefer a simplified risk score predictive model for the sake of simplicity, ease of use in clinical settings, particularly in our country, and improved performance. This could aid in identifying women at higher risk of adverse birth outcomes who require additional management, monitoring, and intervention.

In a study conducted for the prediction of adverse pregnancy outcomes by Medicaid, a prediction model was created with development and validation AUROCs of 0.79 and 0.63, respectively (29). In addition, they validated the model using the sample-split technique, and the performance in validation was lower than the performance in development (the sample sizes were 146 vs. 263). The reason for this could be that sample splitting has limitations due to its inability to quantify the optimism coefficient and the small sample size with many predictors, which results in model over- or underestimation. However, the bootstrapping validation technique was used to validate our model, which measures performance by quantifying the optimism coefficient and also by making the results valid with an adequate sample size (29).

Another study found an AUROC of 0.596 for predicting adverse pregnancy outcomes (60). Internal validation with bootstrapped AUROC was 0.593, and in both development and validation the discrimination performance was poor and the predictors used for the model development were serum biomarkers, including alpha-fetoprotein, human chronic gonadotrophin, and unconjugated estriol. Similarly, in a study conducted in Brazil, the model performance for predicting adverse birth outcomes was 0.699 (41). Our model has higher predictive performance than these studies; this might be due to the fact that the predictors used for model development had a lower contribution to risk discrimination. Furthermore, in the aforementioned studies, the predictors were not easily accessible, needed additional investigation, and were difficult to apply in our setting even if they had good performance.

In a study conducted to validate a risk prediction model for adverse perinatal outcomes in the Netherlands, the development performance was 0.58 and the validation performance was 0.61 (61). Even if the model were valid, its performance in discriminating between low and high risk was poor, but our model has good performance.

The cutoff point for the probability of risk for adverse birth outcomes in our model based on the coefficients was 21% with specificity, sensitivity, negative predictive, and positive predictive values of 83.9%, 67.54%, 90%, and 53%, respectively. The positive likelihood ratio was 4.2 and the negative likelihood ratio was 0.38. The accuracy was 80.3%. In the study conducted by Medicaid, the cutoff point for risk of adverse outcomes was 20% with a sensitivity of 29% and specificity of 82% (29). This finding exhibited lower sensitivity compared to our study, which could result in a higher number of false negatives. This limitation makes it challenging to rule out a high likelihood of an adverse birth outcome (ABO). Consequently, the increased false negatives could significantly hinder the prevention of adverse outcomes, even if the cutoff point is similar to that of our model.

To evaluate the clinical and public health impact of our model, we performed DCA. Even though the model performance was assessed in terms of discrimination and calibration, it may not be used in clinical settings unless it has clinical importance. Our model assumes the referral of pregnant women who are at higher risk of adverse pregnancy outcomes for further management and control. DCA compares the developed model with two extreme scenarios, namely, referring all women and not referring all women regardless of their risk. The developed model had the highest net benefit ratio starting from a threshold probability >0.15 compared to not referring all and referring all regardless of their risk. The model had a similar net benefit to the model referring all regardless of their risk across thresholds ranging from 0 to 0.15. This indicates that a referral decision can be made based on the developed model for optimal treatment and that the model has clinical and public health value.

### Strengths and limitations of the study

The strengths of this study include the fact that we used an adequate sample size (adequate number of participant outcomes) for predicting adverse birth outcomes. Second, the model was developed based on easily accessible and measurable maternal characteristics and is applicable in any clinical setting. Third, the internal validity of the developed model was assessed by bootstrapping. Even though the study was conducted as a follow-up study, there were limitations due to the fact that it was based on secondary data. Some variables were omitted, but they have been reported in the literature as important predictors of adverse birth outcomes. The second limitation was that a mother could be counted twice if she had two pregnancies with different card numbers. There were also missed registrations in the logbooks that were used to create a picture of the study population. The final limitation was that we developed the model in a single area. Thus, we need external validation to confirm the model’s transportability and be able to apply it in other areas, especially primary healthcare institutions.

## Conclusion

This study revealed that the incidence of adverse birth outcomes was high. Residence, hemoglobin level, antepartum hemorrhage, pregnancy-induced hypertension, premature mature rupture of membranes, multiple gestation, and onset of labor were all found to be significant predictors of adverse birth outcomes and were retained in the final reduced model for model development.

The risk prediction model was developed based on readily available maternal characteristics using the original beta coefficients and a simplified risk score. The model had good predictive performance and was well-calibrated in both prediction models. The model was internally valid with a low optimism coefficient. The final model used for risk stratification was a simplified risk score model. A feasible risk score would reduce neonatal morbidity and mortality and improve maternal and child health. The developed model has clinical and public health potential for referring high-risk pregnant women for specific management aimed at promoting favorable birth outcomes.

The risk score for predicting adverse birth outcomes can be integrated into maternal health programs by assessing risk during antenatal care, offering targeted interventions, using digital tools such as mobile health applications, training healthcare workers, and incorporating it into national and global health guidelines to improve maternal and child health outcomes.

Therefore, the predictive model using a simplified risk score will help stratify the risk of ABOs among pregnant women for future management and promote favorable outcomes.

## Data Availability

The original contributions presented in the study are included in the article/Supplementary Material, further inquiries can be directed to the corresponding author.
