# Supplementary material for: Development and validation of a risk score to predict adverse birth outcomes using maternal characteristics in northwest Ethiopia: a retrospective follow-up study
Source: Front Glob Womens Health. 2024 Dec 18;5:1458457. doi: 10.3389/fgwh.2024.1458457 (PMC11688326; doi:10.3389/fgwh.2024.1458457)
Supplement: Supplementary file 1 [file Table1.docx]

**Supplementary tables and figures**

Table 1: Socio-demographic characteristics of pregnant women who had ANC visit at UoGCSH, Ethiopia, 2016-2020 (n=910)

| Variable | Categories | Frequencies | Percentage(%) |
| --- | --- | --- | --- |
| Maternal Age | < 20 | 66 | 7.25 |
|  | 20-34 | 746 | 81.98 |
|  | ≥35 | 98 | 10.77 |
| Residence | Urban | 809 | 88.90 |
|  | Rural | 101 | 11.10 |
| Current Marital status | Un married | 42 | 4.62 |
|  | married | 868 | 95.38 |

*Current marital status unmarried includes pregnant women who had not married (had no husband) in recent pregnancy and includes single, widowed and divorced: married women who had husband during their recent pregnancy.*

Table 2: Maternal reproductive history and birth characteristics of pregnant women who had ANC visit at UoGCSH, 2016-2020

| Variable | Categories | Frequency | Percentage |
| --- | --- | --- | --- |
| Gravidity | Primigravida | 351 | 38.57 |
|  | Multigravida | 559 | 61.43 |
| Parity | Zero | 399 | 43.85 |
|  | Primiparity | 229 | 25.16 |
|  | Multiparty | 282 | 30.99 |
| Past obstetrics outcomes related to foetus | Abortion(n=559) | 106 | 18.9 |
|  | stillbirth(n=511) | 23 | 4.5 |
|  | Early neonatal death(n=511) | 25 | 4.89 |
|  | Low birthweight (n=511) | 2 | 0.4 |
|  | preterm (n=511) | 2 | 0.4 |
| Modern Family planning use | No | 430 | 47.25 |
|  | Yes | 480 | 52.75 |
| Types of family planning used | Injectable | 252 | 52.5 |
|  | Pills | 156 | 32.5 |
|  | Implanon | 63 | 13.12 |
|  | Others* | 9 | 1.87 |

*Others* includes family planning used emergency contraceptive and intrauterine contraceptive device*

Table 3: Medical illness of pregnant mother who had ANC visit at UoGCSH, 2016-2020

| Variables | Categories | Frequency | Percentage |
| --- | --- | --- | --- |
| Medical illness | No | 817 | 89.78 |
|  | Yes | 93 | 10.22 |
| Types of medical illness | HIV/AIDS | 31 | 33.3 |
|  | UTI | 59 | 63.4 |
|  | Chronic hypertension | 5 | 5.4 |
|  | Others | 20 | 21.5 |

*medical illness includes HIV/AIDS: Human Immune Deficiency Virus/ Acquired Immune Deficiency Virus, UTI: Urinary Tract Infection, chronic hypertension and others: other types of medical illnesses include hepatitis b, cardiac illness, kidney disease(nephrolithiasis), thyrotoxicosis, hypothyroidism and diabetes mellitus*

Table 4:Antenatal characteristics of pregnant women in their recent pregnancy who attend ANC unit at the UOGCSH, 2016-2020

| Variable | Categories | Frequency | Percentage |
| --- | --- | --- | --- |
| Maternal haemoglobin level (g/dl) | Normal | 835 | 91.76 |
|  | low | 75 | 8.24 |
| Timing of ANC initiation | 1st trimester | 77 | 8.46 |
|  | 2nd trimester | 491 | 53.96 |
|  | 3rd trimester | 342 | 37.58 |
| Number of ANC visits | 1 | 53 | 5.82 |
|  | 2-3 | 286 | 31.43 |
|  | ≥4 | 571 | 62.75 |
| Iron folate supplementation | No | 89 | 9.78 |
|  | Yes | 821 | 90.22 |
| Time of initiating iron folate | 1^st^ trimester | 12 | 1.46 |
|  | 2^nd^ trimester | 385 | 46.78 |
|  | 3^rd^ trimester | 426 | 51.76 |
| Pregnancy status | Planned and wanted | 857 | 94.18 |
|  | Unplanned but wanted | 30 | 3.30 |
|  | Unplanned Unwanted | 23 | 2.53 |
| Nutritional advices | No | 376 | 41.32 |
|  | Yes | 534 | 58.68 |
| Rh factor | Positive | 840 | 92.31 |
|  | negative | 70 | 7.69 |

*ANC: Antenatal Care, Rh factor- rhesus factor*

Table 5:Recent pregnancy complication, labor and delivery characteristics pregnant women who had ANC visit at UOGCSH, 2016-2020

| Variables | categories | Frequencies | Percentage |
| --- | --- | --- | --- |
| Recent pregnancy complication | No | 708 | 77.80 |
|  | Yes | 202 | 22.20 |
| Types of recent pregnancy complication | PIH | 74 | 36.6 |
|  | APH | 57 | 28.2 |
|  | PROM | 67 | 33 |
|  | Others | 83 | 41 |
| Number of foetus | Singleton | 888 | 97.58 |
|  | Multiple | 22 | 2.42 |
| Presentation of foetus | Normal | 866 | 95.16 |
|  | Malpresentation | 44 | 4.84 |
| Onset of foetus | Induced | 94 | 10.33 |
|  | Spontaneous | 816 | 89.67 |
| Mode of mode of delivery | Spontaneous | 634 | 69.7 |
|  | caesarean section | 226 | 24.84 |
|  | Instrumental | 50 | 5.49 |

*APH antepartum hemorrhage, PIH pregnancy induced hypertension, PROM premature rupture of membrane, other recent pregnancy complication includes (Amniotic Fluid index (oligohydramnios and polyhydramnios) gestational diabetes mellitus and chorioaminties), Malpresentation presentation of fetus other than vertex, GDM gestational diabetic mellitus:*

Table 6: The incidence of adverse birth outcomes among pregnant women who had ANC visit at UOGCSH from 2016-2020

| Birth outcome | Categories | Number | Percentage |
| --- | --- | --- | --- |
| Adverse birth outcome | No | 715 | 78.57 |
|  | Yes | 195 | 21.43 |
| Status of new born | Live birth | 863 | 94.84 |
|  | Stillbirth | 47 | 5.16 |
| Birth weight(in gm) | <2500 | 113 | 12.42 |
|  | ≥2500 | 797 | 87.58 |
| Gestational age at time of delivery | Preterm | 108 | 11.87 |
|  | Term | 802 | 88.13 |
| Congenital malformation | No | 895 | 98.35 |
|  | Yes | 15 | 1.65 |

*Preterm birth; is birth before 37 completed weeks, congenital malformation visible congenital defect including ancephaly, ecephlocele, hydrocephalus, ventricular defect, cleft pallet, and clubbing of foot.*

Table 7: The univariable analysis for Adverse birth outcomes among pregnant women who had ANC visit at UoGCSH, 2016-2020

| Variables | Coefficients with 95%CI | P_value |
| --- | --- | --- |
| Age  20-34 | 0.19(-0.45, 0 .84) | 0.567 |
| >35 | 0.43(-0.34, 1.21) | 0.273 |
| Marital status (married) | 0.023(0-.77, 0.82) | 0.954 |
| Residence | 1.1 (0.63, 1.50) | <0.001 |
| Medical illness (yes) | 0.63(0.16 ,1.1) | 0.008 |
| Family planning history(no) | .10(-.215, .417) | 0.533 |
| Pregnancy status (unplanned) | .049(-0.61,0 .71) | 0.883 |
| Time initiating ANC (1^st^ tm ref) |  |  |
| Second trimester | -.22(-.80, .34) | 0.437 |
| Third trimester | .017(-.56, .60) | 0.954 |
| Number of ANC( >4 refer) |  |  |
| 1 time | .20(-0.45,0.85) | 0.551 |
| 2-3 times | .039(-0.306, 0.38) | 0.823 |
| Gravidity(multigravida) | .033 (-0.29, 0.36) | 0.840 |
| GDM(yes) | .096(1.20, 1.39) | 0.004 |
| AFI(normal ref) |  |  |
| Oligohydramnios | .087( -0.74, .56) | 0.794 |
| Polyhydramnios | .45(-0.91, 1.81) | 0.516 |
| APH(yes) | 1.8(1.29,2.405) | <0.001 |
| PIH(yes) | 2.0(1.52,2.526) | <0.001 |
| PROM(yes) | 1.5 (1.03, 2.05) | <0.001 |
| Haemoglobin(low) | 1.30(0.820,1.79) | <0.001 |
| Number of foetus(multiple) | 2.13 (1.21, 3.04) | <0.001 |
| Presentation (abnormal) | 1.19(0.57,1.80) | <0.001 |
| Labor onset(induced) | 1.35 (.913, 1.796) | <0.001 |
| Mode of delivery(ref SVD) |  |  |
| Instrumental | .106(-0.82, 0.61) | 0.770 |
| Caesarean section | .057(-0.43, 0.31) | 0.761 |
| Prolonged labor(yes) | .247 (0.081, 0.576) | 0.040 |

*CI confidence interval, SVD spontaneous vaginal delivery, AFI amniotic fluid index*

Table 8: Multi-variable binary logistic regression coefficients and risk score for variables retained in the final reduced model for prediction of adverse birth outcome among pregnant women attending ANC , 2016-2020.

| Predictor | Adverse birth outcome | | Multivariable analysis | | Simplified risk score |
| --- | --- | --- | --- | --- | --- |
|  | Yes | No | β(95 % CI) | p_value |  |
| **Residence**  Urban  Rural | 154  41 | 655  60 | 0  0.937(0.42, 1.43) | 0.00028 | 1 |
| **APH**  No  Yes | 161  34 | 692  23 | 0  1.651(1.024, 2.28) | 0.0002719 | 2 |
| **PIH**  no  yes | 149  46 | 687  28 | 0  1.761(1.198, 2.33) | 0.00108 | 2 |
| **HGB level**  normal low | 160  35 | 675  40 | 0  1.372(0.82, 1.92) | 0.00957* | 2 |
| **PROM**  No  Yes | 160  35 | 683  32 | 0  1.295(0.70, 1.88) | 0.0000148 | 2 |
| **N_Fetus**  Singleton  Multiple | 168  17 | 710  5 | 0  2.551(1.5, 3.73) | 0.00036 | 3 |
| **Onset of labour**  Spontaneous  Induced | 151  44 | 665  50 | 0  **0.8184**(0.28,1.34) | 0.00232 | 1 |
| Intercept |  |  | -2.23876 |  | 13 total risk score |

*APH antepartum hemorrhage, PIH pregnancy induced hypertension, HGB hemoglobin, PROM premature rapture of membrane, N_Fetus -number of fetus during recent pregnancy, simplified risk score we divided the coefficient of predictors in the final reduced model to lowest coefficient which is 0.8184 and rounding to nearest integer, CI confidence interval*

Table 9: Performance of the predicted model based on original beta coefficients at different cut off points for pregnant women who had ANC at UOGCSH, 2016-2020.

| Cut-off point | Sensitivity | Specificity | PPV | NPV | Accuracy |
| --- | --- | --- | --- | --- | --- |
| 0.1536 | 76 | 74 | 44 | 92 | 74 |
| 0.2000 | 70 | 80 | 48 | 90.8 | 77 |
| **0.2139** | **67.54** | **83.9** | **53** | **90** | **80** |
| 0.416 | 50.2 | 94 | 70 | 87 | 84 |

Table 10: Risk classification for adverse birth outcome based on simplified risk score (n=910) among pregnant women who had ANC at UOGCSH, 2016-2020

| Risk score categories | Prediction model based on Maternal characteristics | |
| --- | --- | --- |
|  | Total number of women | Incidence of ABO |
| Low risk(<2) | 608(66.8%) | 55(9.04%) |
| Intermediate(2-4) | 211(23.18%) | 74(35.1%) |
| High risk (≥4) | 91(0.1%) | 66(72.5%) |
| Total | 910(100%) | 195(21.43%) |

*Risk score =1*residence(rural)+ (2*APH (yes)) +(2 *PIH(yes)) + (2*Hemoglobin(low)) + (2*PROM(yes)) +(3*N_Fetus(multiple)) + (1*labor (induced))*

Table 11: Performance of risk score at different cutoff point for adverse birth outcome among pregnant women attending antenatal care at UOCSH , 2016 to 2020

| cut-off | High risk n(%) | Sensitivity | Specitity | PPV | NPV | LP+ | LR- |
| --- | --- | --- | --- | --- | --- | --- | --- |
| 1 | 338(37.1) | 73.8 | 72.8 | 42.6 | 91.08 | 2.71 | 0.359 |
| **2** | **302(33.18)** | **71.79** | **77.34** | **46.35** | **90.95** | **3.16** | **0.365** |
| 3 | 184(20.2) | 50.25 | 87.97 | 53.26 | 86.6 | 4.177 | 0.56 |
| 4 | 91(10) | 33.84 | 96.5 | 72.52 | 84.24 | 9.666 | 0.686 |
| 5 | 66(7.25) | 25.12 | 97.62 | 74.24 | 82.70 | 10.55 | 0.76 |

*PPV positive predictive value, NPV negative predictive value, LR+ likelihood ratio positive, LR- likelihood ratio negative*


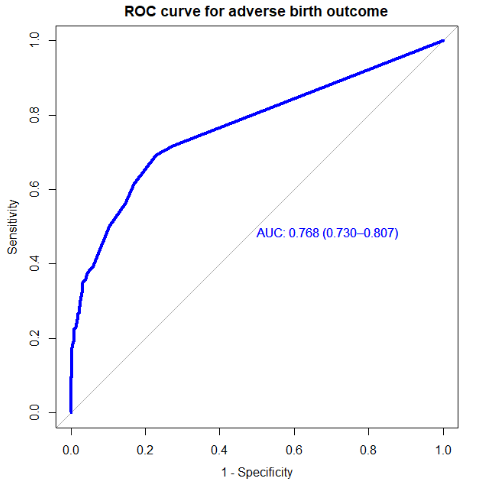


Figure1: The ROC curve represents the probability of risk for adverse birth outcome among women who had ANC visit at UOGCSH, 2016-2020


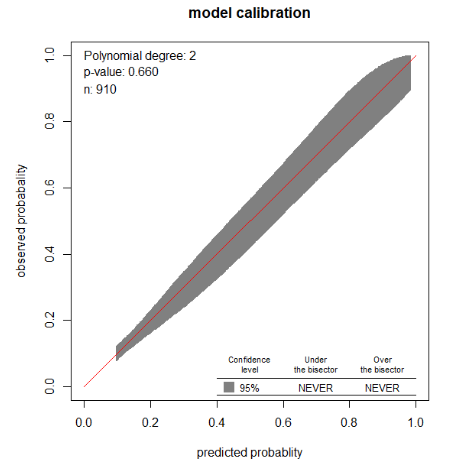


Figure 2: Calibration plot for developed model based on original beta coefficient for risk prediction model for pregnant women who had ANC at UOGCSH, 2026-2020.


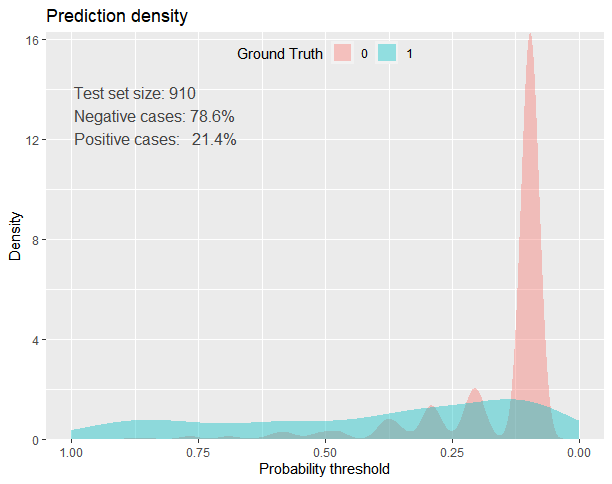


Figure 3: Prediction density plot for developed model using original beta coefficients at UOGCSH, 2016-2020.

ple) + 1*labor onset(yes)


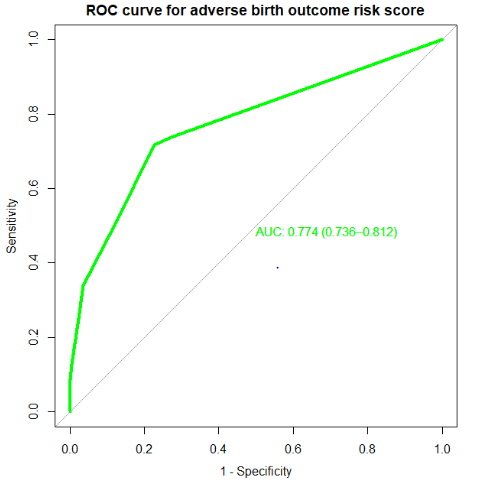


Figure 4: Area under the ROC curve for the prediction mode for ABO using simplified risk score.


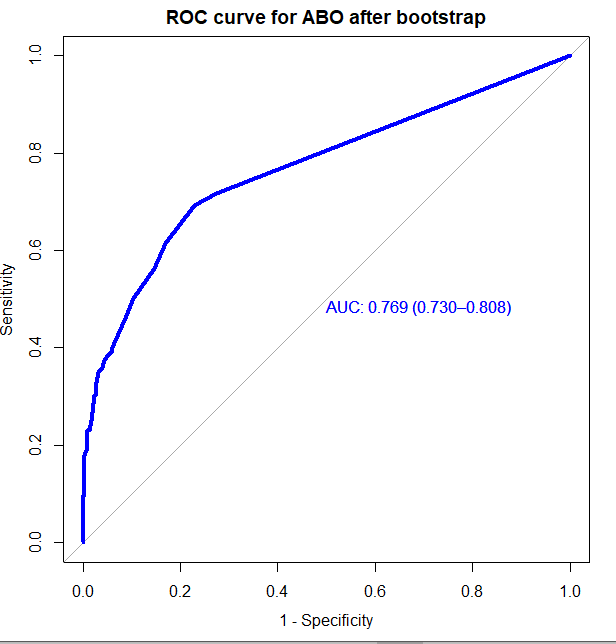


Figure 5: Area under the ROC curve for the prediction model for bootstrapped sample, UOGCSH, 2016-2020


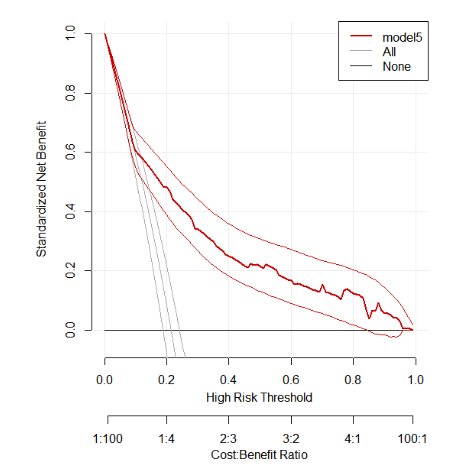


Figure 6: Decision curve analysis curve of the developed model plotted net benefit vs threshold probability between model and two extreme scenarios at UOGCSH, 2016-2020
